# Cork-in-bottle mechanism of inhibitor binding to mammalian complex I

**DOI:** 10.1126/sciadv.abg4000

**Published:** 2021-05-14

**Authors:** Injae Chung, Riccardo Serreli, Jason B. Cross, M. Emilia Di Francesco, Joseph R. Marszalek, Judy Hirst

**Affiliations:** 1 MRC Mitochondrial Biology Unit, University of Cambridge, The Keith Peters Building, Cambridge Biomedical Campus, Hills Road, Cambridge CB2 0XY, UK.; 2 Institute for Applied Cancer Science (IACS), The University of Texas MD Anderson Cancer Center, Houston, TX 77054, USA.; 3 TRACTION–Translational Research to AdvanCe Therapeutics and Innovation in ONcology, The University of Texas MD Anderson Cancer Center, Houston, TX 77054, USA.

## Abstract

Mitochondrial complex I (NADH:ubiquinone oxidoreductase), a major contributor of free energy for oxidative phosphorylation, is increasingly recognized as a promising drug target for ischemia-reperfusion injury, metabolic disorders, and various cancers. Several pharmacologically relevant but structurally unrelated small molecules have been identified as specific complex I inhibitors, but their modes of action remain unclear. Here, we present a 3.0-Å resolution cryo–electron microscopy structure of mammalian complex I inhibited by a derivative of IACS-010759, which is currently in clinical development against cancers reliant on oxidative phosphorylation, revealing its unique cork-in-bottle mechanism of inhibition. We combine structural and kinetic analyses to deconvolute cross-species differences in inhibition and identify the structural motif of a “chain” of aromatic rings as a characteristic that promotes inhibition. Our findings provide insights into the importance of π-stacking residues for inhibitor binding in the long substrate-binding channel in complex I and a guide for future biorational drug design.

## Teaser

A multidisciplinary approach reveals a unique mechanism of mammalian complex I inhibition relevant for cancer therapeutics.

## INTRODUCTION

Mitochondrial complex I {NADH [reduced form of nicotinamide adenine dinucleotide (NAD^+^)]:ubiquinone oxidoreductase} (*1*, *2*) is an essential enzyme for mitochondrial energy metabolism that couples oxidative phosphorylation for adenosine 5′-triphosphate (ATP) synthesis to regeneration of NAD^+^ for the tricarboxylic acid cycle and fatty acid oxidation. While specific mutations in complex I genes cause a host of primary mitochondrial disorders, complex I is further implicated in common pathologies such as ischemia-reperfusion (IR) injury [initiated by a burst of reactive oxygen species (ROS) generated at complex I via reverse electron transport (RET)], metabolic disorders including insulin resistance (*3*), and subsets of cancers reliant on oxidative phosphorylation (*4*). Complex I is therefore increasingly recognized as a promising target for therapeutic intervention through the development of pharmacologically relevant inhibitors of the enzyme. The S1QEL (suppressors of site I_Q_ electron leak) inhibitors (*5*) are reported to block ROS production at complex I during RET, and mitochondria-targeted thiol species that react with the deactive state of complex I formed during ischemia have been shown to be protective against IR injury (*6*). Metformin, a widely used drug for type 2 diabetes (*7*), is known to inhibit complex I, and the anthelmintic reagent nafuredin also targets complex I (*8*). A range of complex I inhibitors have also been investigated and developed as anticancer compounds, including BAY 87-2243 (*9*), IACS-010759 (*10*), mubritinib (*11*), carboxyamidotriazole (*11*), deguelin (*12*), hydroxylated rotenoids (*13*), and quinazoline diones (*14*), although often with limited knowledge about their modes of actions. At the same time, complex I is also a major contributor to drug-induced mitochondrial dysfunction, for example, by antipsychotics (*15*), through increased oxidative stress, impaired ATP synthesis, or imbalances in the NADH/NAD^+^ ratio (*16*).

Many inhibitors of complex I are assumed to bind in the unusually long and narrow channel that leads “upward” from the membrane plane, in which its highly hydrophobic substrate ubiquinone-10 is proposed to bind. This elongated tunnel is hydrophobic at both ends but charged and polar in the center (*17*, *18*) because of a network of charged residues that may be involved in propagating energy liberated by quinone-mediated redox catalysis to proton pumping (*19*–*22*). So far, the ubiquinone-analog complex I inhibitor piericidin A1 has been observed to bind at the top of the channel, adjacent to the two proposed ligands of the ubiquinone headgroup, NDUFS2 His59 and Tyr108 in the mammalian complex (*23*) and in *Thermus thermophilus* (*24*). Aureothin and pyridaben, which are also quinone-like compounds, were likewise observed to bind at a similar site in *T. thermophilus* (*24*). The headgroups of the inhibitor 2-decyl-4-quinazolinyl amine (DQA) (*19*) and the detergent *n*-dodecyl β-d-maltoside (DDM) (*25*) have been observed to bind partway up the channel in the aerobic yeast *Yarrowia lipolytica*, while rotenone has been observed to bind at both sites in the channel in addition to a third site in the antiporter-like subunit ND4 (*26*). However, no structures of complex I bound to pharmacologically relevant inhibitors, which typically bear little or no resemblance to ubiquinone (*5*, *7*, *9*, *11*, *14*), are currently available. Structures with noncanonical bound inhibitors offer untapped possibilities to reveal functionally relevant features of complex I and key features of inhibitory modes of action, as well as to establish the significance of chemical substructures or skeletons as promising seeds for advancements in biorational drug design.

Recently, the keyhole-lock-key model has been proposed for catalysis by enzymes with elongated cavities or “tunnels” that lead to a buried active site, where the substrate (key) must pass through a tunnel (keyhole) to reach the active site (lock) to be converted into the product (*27*, *28*). This model implies that the access tunnels are important structural features that could influence the transfer of and specificities for the substrates and products, with particular significance resting on the tunnel length and geometry, entrance and bottleneck radius and residues, and protein dynamics (*27*, *28*). Specific inhibitors may bind in the tunnel, interacting with tunnel-lining residues and blocking the tunnel as a transport pathway (*28*). Intrinsically, this proposed model applies to the long ubiquinone-access tunnel of mitochondrial complex I, which exhibits a diversity of environments that may explain why so many structurally unrelated diverse small-molecule natural products and synthetic compounds are inhibitors of it (*29*, *30*) and why drug design and prediction of toxicity are so challenging. However, only very limited structural information is available on any inhibitor-bound states.

IACS-010759 is a highly potent and selective small-molecule inhibitor of complex I that was developed by the MD Anderson Cancer Center (*10*) and found through lead optimization seeded with known modulators of hypoxia-inducible factor 1-α that act via inhibition of oxidative phosphorylation (*9*, *31*, *32*). It contains a heterocyclic 1,3-nitrogen motif, a structural motif also present in two other anticancer therapeutic drugs, which are proposed to act through complex I inhibition (*11*). IACS-010759 robustly reduced cell proliferation and increased apoptosis at well-tolerated doses in both in vitro and in vivo models of brain cancer, acute myeloid leukemia (AML) (*10*), and Bruton’s tyrosine kinase inhibitor ibrutinib-resistant mantle cell lymphoma (*33*), all of which are dependent on oxidative phosphorylation because they have reduced compensatory glycolytic capacity for maintaining ATP levels. IACS-010759 is currently being evaluated in phase 1 clinical trials in subjects with relapsed/refractory AML (NCT02882321), as well as for solid tumors and lymphoma (NCT03291938). Metabolomic analyses suggested that the IACS-010759–mediated effects result from a combination of energy depletion and reduced aspartate production that leads to impaired nucleotide biosynthesis (*10*), and initial biochemical investigation has established IACS-010759 as an ubiquinone-binding site complex I inhibitor, with no effect on flavin site activity or ROS production from complex I (*10*). It has been proposed that the inhibitor binds in or at the entrance to the ubiquinone-binding cavity as an L55F mutation close to the entrance of the channel (in the membrane-embedded ND1 subunit of H292 clonal cell lines) reduced sensitivity to IACS-010759 (*10*). Alternatively, photoaffinity labeling studies by Tsuji *et al.* (*34*) suggested that IACS-010759 does not occupy the ubiquinone-binding site but rather indirectly affects the quinone redox reactions by inducing structural changes of the ubiquinone-binding channel by binding to the region under the matrix-side loop that connects transmembrane helix 5 (TMH5) to TMH6 of the ND1 subunit.

Here, we have used IACS-2858, a more potent close structural analog of IACS-010759, to determine the first structure of mammalian complex I bound by a noncanonical, pharmacologically relevant inhibitor. Our structure provides a clear molecular understanding of the interaction and mechanism of action of the anticancer drug candidate IACS-010759 (*10*). Integrating structural and kinetic approaches, we delineate mechanistically crucial enzyme-inhibitor contact sites across species and additionally find evidence against a second putative ubiquinone-binding entrance. Our IACS-2858–bound structure therefore presents insights into the inhibitory mechanism of a complex I inhibitor that bears little resemblance to its native substrate ubiquinone. Our data are relevant to complex I–focused drug design strategies, understanding complex I–mediated drug toxicity and highlight hitherto overlooked functionally relevant features of the ubiquinone-binding channel.

## RESULTS

### Determination of the cryo–electron microscopy density map for IACS-2858–inhibited mouse complex I

**Fig. 1:**
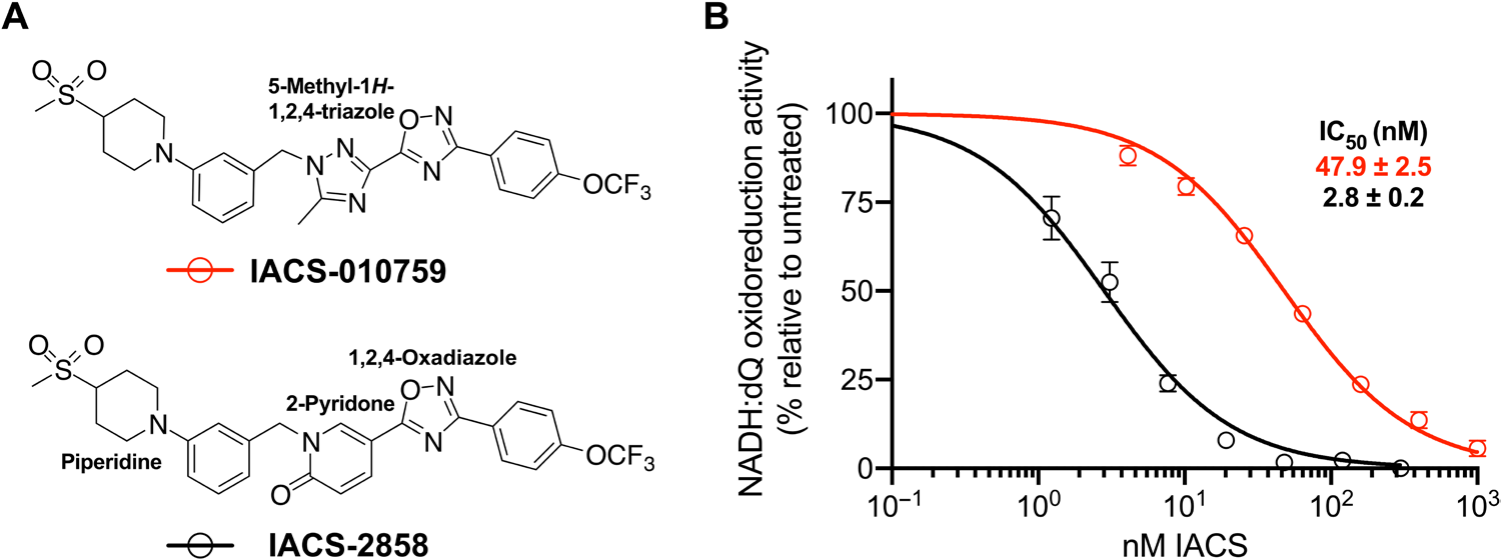
IACS-2858 is a stronger inhibitor of mouse complex I than IACS-010759. (**A**) Chemical structures of IACS-010759 and IACS-2858. (**B**) Experimentally measured NADH:decylubiquinone (dQ) oxidoreduction rates of purified mouse complex I (average ± SEM), technical replicates (*n* ≥ 3) plotted against a titration of IACS-010759 (red) or IACS-2858 (black) concentrations. Hill slopes were constrained to −1. IC_50_ values are reported with the SE.

Two candidates from the IACS-010759 family were evaluated for their application in structural studies by testing their ability to inhibit isolated complex I from mouse heart mitochondria (Fig. 1). The median inhibitory concentration (IC_50_) value (concentration required for 50% inhibition, under the conditions specified) obtained for IACS-010759 itself was 47.9 nM, defining it as a good inhibitor of mouse complex I, but not a tight-binding one, given that the complex I concentration in the assay was ~0.5 nM. Therefore, we chose to work instead with IACS-2858, a closely related variant with an IC_50_ value of 2.8 nM (under the same conditions), to optimize inhibitor occupancy of the active site in cryo–electron microscopy (cryo-EM) analyses. Like IACS-010759, IACS-2858 has a five-ring skeleton consisting of four aromatic and one nonaromatic rings flanked on each end by methylsulfonyl and trifluoromethoxy groups. The sole difference is that IACS-2858 has a 2-pyridonyl ring in lieu of the 5-methyl-1*H*-1,2,4-triazole group of IACS-010759.

IACS-2858–inhibited mouse complex I was purified using a protocol adapted from that used previously to purify the piericidin A1–bound enzyme (*23*). Key features were addition of saturating IACS-2858 as a dry solid to avoid the addition of an organic solvent, followed by size exclusion chromatography to remove any free or nonspecifically bound inhibitor. The extent of inhibition of the isolated IACS-2858–bound enzyme was 81 ± 5% for the sample used for cryo-EM analyses, by comparing its initial turnover rate with that of a matching control sample prepared without inhibitor. Three cryo-EM datasets were collected on two grids prepared from the same sample. They were analyzed independently using RELION-3.0.7, combined in RELION-3.1, and reached a global resolution of 3.0 Å (table S1 and fig. S1) (*23*). Local resolution estimates of the electrostatic potential map and half-map validations are shown in fig. S2. The map was modeled starting from a model for the active state of mouse complex I (*23*) and summarized in table S2.

The global conformation of the structure conforms closely to the cryo-EM map and model for the active state of mouse complex I (*23*) {97% correlation between the IACS-2858–bound and active maps [Electron Microscopy Data (EMD): 11377] in UCSF ChimeraX and a global all-atom root mean square deviation (RMSD) of 0.59 Å [Protein Data Bank (PDB): 6ZR2] for all atoms, relative to 83% and 2.31 Å for the deactive state (EMD: 4356; PDB: 6G72) (*20*), respectively}. Hallmarks of the active state of complex I (*20*, *23*) including observable densities for the loops in NDUFS2, ND3 and ND1; an extended interface between NDUFA5 and NDUFA10 (*20*); and the lack of a π-bulge in TMH3 of ND6 observed in the deactive state are conserved in the IACS-2858–inhibited enzyme. Subsequently, detailed comparisons of the IACS-2858–inhibited model with the active enzyme model (*23*) did not identify any material differences, showing that IACS-2858 binds to the active state of the enzyme that contains a structured ubiquinone-binding site.

### Cork-in-bottle mechanism of inhibition by IACS-2858

**Fig. 2:**
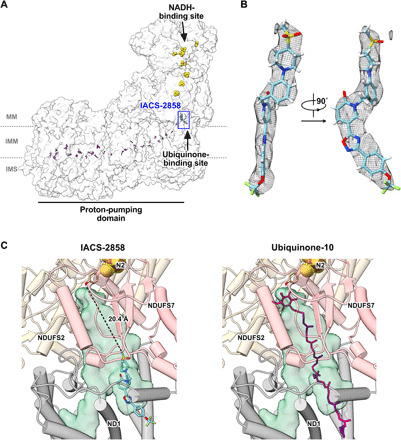
IACS-2858 bound in the structure of mouse complex I. (**A**) A single IACS-2858 molecule (blue) binds at the entrance of the ubiquinone-binding site. The iron-sulfur clusters (red and yellow) and the ubiquinone-binding site are indicated, and charged residues in the proton-transfer domain are marked in purple. MM, mitochondrial matrix; IMM, inner mitochondrial membrane; IMS, intermembrane space. (**B**) Electrostatic potential density of IACS-2858, presented from two viewpoints and plotted using UCSF ChimeraX with contour level 0.05. (**C**) IACS-2858 (blue) in the ubiquinone-binding cavity formed by the surrounding subunits (left) and the expected position of ubiquinone-10 (magenta; alternating isoprenoid units in purple) (*17*) modeled into the cavity (right). The interior surface cavity in green was identified using CASTp (*57*).

The cryo-EM Coulomb potential map of the IACS-2858–bound enzyme shows an unambiguous density for IACS-2858 in the entrance of the ubiquinone-binding site (Fig. 2), overlapping the location where isoprenoids 4 to 9 of the ubiquinone-10 tail are predicted to be located (*17*) when ubiquinone-10 is fully bound with its headgroup in the active site. The resolution of the map feature allows the orientation of the inhibitor in the site (i.e., which end enters first) to be clearly identified. The structure of IACS-2858 bound to complex I thereby provides a simple explanation for its mechanism of action: IACS-2858 behaves like a cork and plugs the entrance of the ubiquinone-binding channel (Fig. 2C). The terminal sulfonylmethyl group of the inhibitor, which extends furthest into the channel, is located 20.4 Å away from Tyr108^NDUFS2^, one of the two proposed ligands of the ubiquinone headgroup in the active site for ubiquinone reduction.

### The coordination of enzyme-bound IACS-2858

**Fig. 3:**
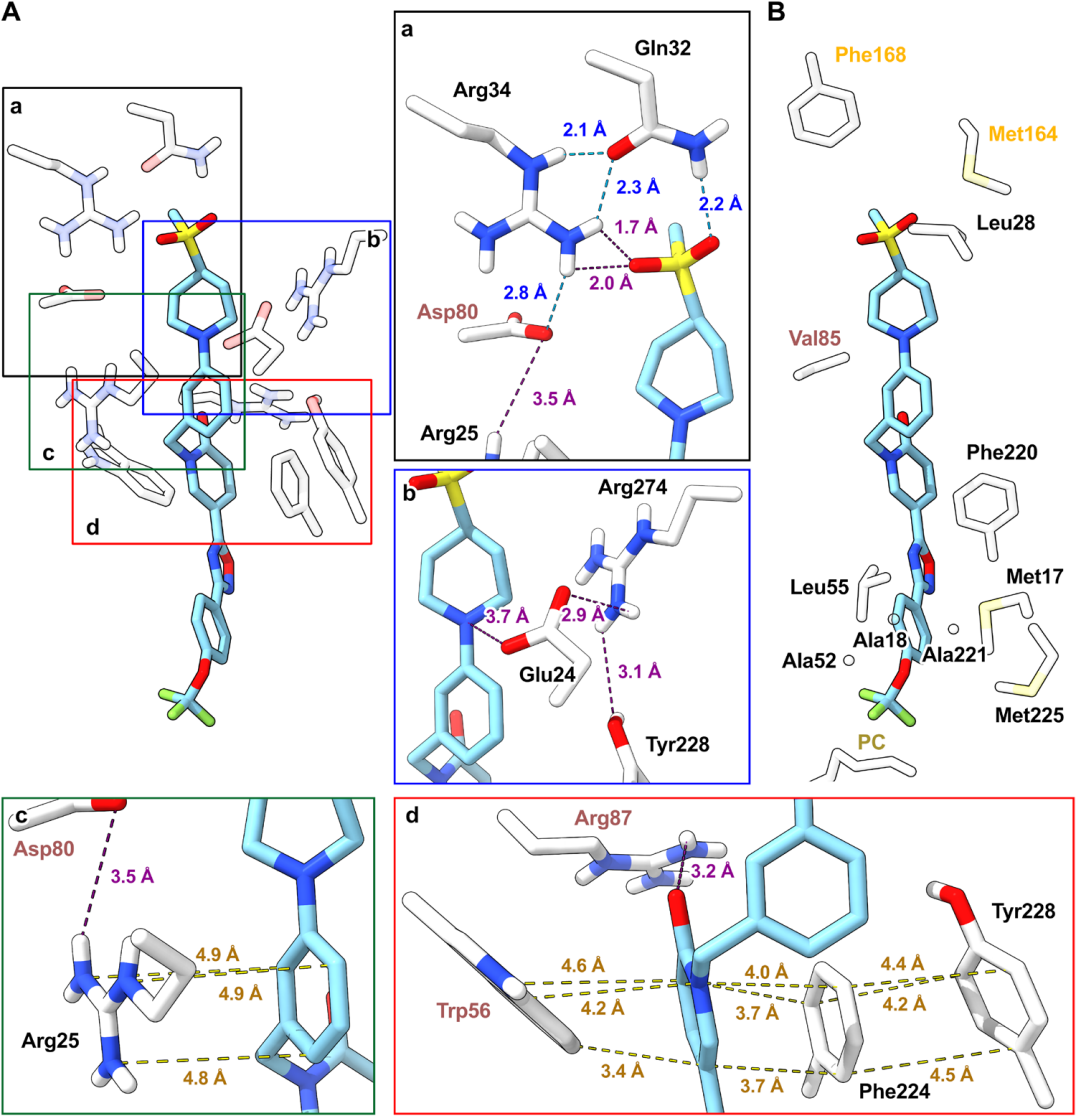
IACS-2858 interacts with residues at the entrance of the ubiquinone-binding site. (**A**) Hydrogen bonding (blue dashed line) and π-stacking (yellow dashed line) and polar interactions (purple dashed line) made between IACS-2858 (sky blue) and residues (white) surrounding the (a) sulfonylmethyl (black box), (b) piperidine (blue box), (c) phenyl (green box), and (d) 2-pyridone (red box) moieties of IACS-2858. Insets a to d have been marginally rotated to give the clearest field of view. (**B**) Van der Waal’s and hydrophobic contacts made by residues within 3.5 Å of IACS-2858. Residues from ND1, NDUFS2, and NDUFS7 are labeled in black, yellow-orange, and pink, respectively. PC, phosphatidylcholine.

Figure 3 shows the residues that interact with IACS-2858 in the ubiquinone-binding pocket of mouse complex I and form a total of 110 atomic contacts with it. In the central charged region of the ubiquinone-binding pocket where the fourth isoprenoid unit of the tail is expected to lie (*17*), a triad of polar residues (Gln32^ND1^, Arg34^ND1^, and Asp80^NDUFS7^) in a hydrogen-bonding network point into the cavity, such that the amine group of Gln32^ND1^ forms a hydrogen bond to one of the two IACS-2858 terminal sulfonyl oxygens, and the terminal amine of Arg34^ND1^ contributes further polar interactions (Fig. 3A, a). Other residues within 3.5 Å of the sulfonylmethyl group include Phe168^NDUFS2^, Met164^NDUFS2^, and Leu28^ND1^ (Fig. 3B). Around the piperidine and phenyl rings (see Fig. 1A for the structure of IACS-2858), the side chains of Asp80^NDUFS7^, Arg274^ND1^, Tyr228^ND1^, and Val85^NDUFS2^ are positioned for van der Waals interactions (< 3.5 Å) [Fig. 3, A (a and b) and B], while Glu24^ND1^ makes polar contacts with both the piperidine nitrogen and neighboring Arg274^ND1^ (Fig. 3A, b). The phenyl ring is further involved in π-stacking interactions with the resonant guanidino side chain of Arg25^ND1^, which also forms polar contacts with Asp80^NDUFS7^ (Fig. 3A, c), one of the triad of polar residues described above (Fig. 3A, a). IACS-2858 is sharply twisted at its most mobile region, the methylene group between the phenyl and 2-pyridonyl rings (an sp^3^ carbon center, which allows rotation around its C─C and C─N bonds) and clamped tightly in place also by π-stacking between the 2-pyridone and the aromatic side chains of Phe224^ND1^ and Trp56^NDUFS7^ (Fig. 3A, d). A further π-stacking interaction between Phe224^ND1^ and Tyr228^ND1^ creates a notable series of four successive π-stacking aromatic rings (Fig. 3A, d). These π-interactions are consistent with an “off-center parallel” stacking arrangement.

In addition to the π-stacking interactions, the 2-pyridone is further stabilized at its carbonyl group by polar interactions with Arg87^NDUFS7^ (Fig. 3A, d) or perhaps via hydrogen bonding with the same residue mediated by an intervening water molecule, for which there is a putative unmodeled density. The interaction with Arg87^NDUFS7^ neatly explains the observed structure-activity relationship between IACS-2858 and IACS-010759 because IACS-010759, which has a 17-fold higher IC_50_ value for catalysis by isolated mouse complex I than IACS-2858 (Fig. 1B), has a 5-methyl-1*H*-1,2,4-triazole instead of the 2-pyridonyl moiety (Fig. 1A). It thus lacks the carbonyl and its stabilizing interactions with Arg87^NDUFS7^. Approaching the entrance of the ubiquinone-binding channel (Fig. 2C), the 1,2,4-oxadiazole and phenyl rings of IACS-2858 are surrounded by small or hydrophobic residues of the ND1 subunit (Met17, Ala18, Ala52, Leu55, Ala221, and Met225) (Fig. 3B). Intriguingly, Leu55^ND1^, which is positioned in close proximity (2.8 Å) to the 1,2,4-oxadiazole group, was identified previously as important for inhibition by IACS-010759 because the Phe variant in H292 cells is substantially less sensitive to it (*10*). Last, at the mouth of the channel, the trifluoromethoxy terminus of the inhibitor protrudes into the hydrophobic phospholipid/detergent belt. Notably, the fatty-acid tail of a phosphatidylcholine modeled in several other structures (*20*, *23*, *35*) on the hydrophobic surface outside the channel entrance approaches to within 2.7 Å of one of the terminal fluorines (Fig. 3B).

### Cross-species differences in inhibitor kinetics

**Fig. 4:**
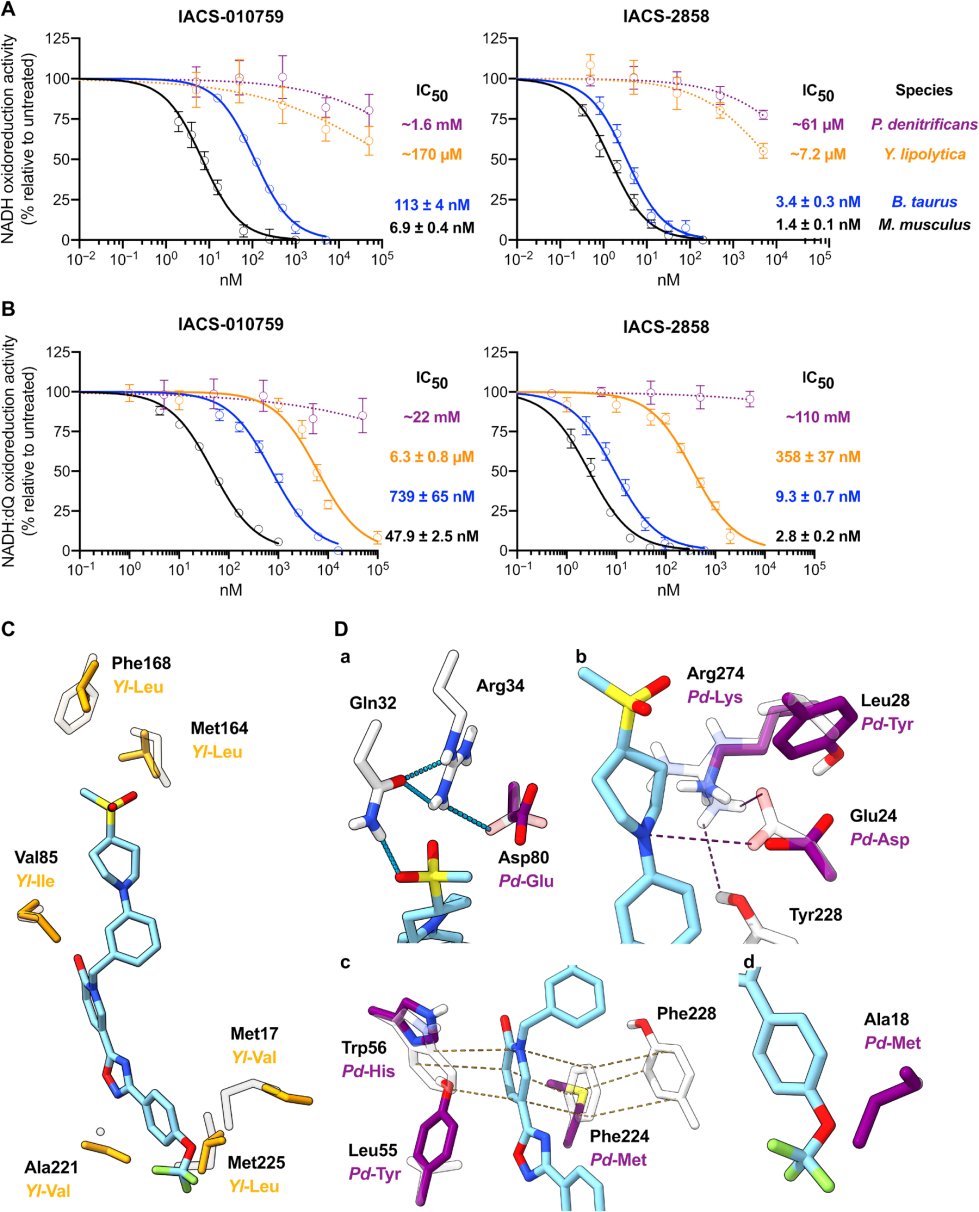
IACS-2858 binds to mammalian, yeast, and bacterial complexes I with different affinities. Experimentally measured NADH oxidation rates (average ± SEM, *n* ≥ 3) in (**A**) membranes or (**B**) purified complex I plotted against inhibitor concentration. IC_50_ values for each inhibitor and species pair are indicated on the right with the SE. Hill slopes were either fixed to −1 (solid lines) or not constrained (dashed lines). (**C**) An overlay of the structures of wild-type *Y. lipolytica* respiratory complex I (PDB: 6YJ4) (*25*) and the IACS-2858–bound mouse complex I showing residues within 3.5 Å of IACS-2858 that are not conserved. (**D**) Details of an overlay of the IACS-2858–bound complex I model with a hypothetical model for *P. denitrificans* complex I, created by mutating the mouse model in PyMOL. a to d show specific regions where differences in residues affect key interactions or result in likely steric clashes. Inhibitor interactions determined in the IACS-2858–inhibited model are indicated as in Fig. 3. The models (except for *M. musculus* in white) are colored by species as in (A) and (B). Residues are labeled for *M. musculus* and mutations to them noted in color accordingly.

IACS-010759 was reported previously to exhibit up to 230-fold variation in IC_50_ across several human cell line models and representative cell lines from mammalian species widely used for preclinical safety analysis, including mouse, monkey, dog, and rat (*10*). The variation contrasts with the expected similar potency of well-characterized inhibitors of complex I, such as piericidin A and rotenone, across all mammalian species. Aided by our clear understanding of how IACS-2858 (a representative member of the IACS-010759 family) interacts with mouse complex I (Fig. 3), we decided to investigate this phenomenon further by expanding our search to include two mammalian (*Mus musculus* and *Bos taurus*), a yeast (*Y. lipolytica*), and a bacterial (*Paracoccus denitrificans*) species. IC_50_ values were thus determined for IACS-010759 and IACS-2858 during NADH oxidoreduction in mitochondrial/bacterial membranes (keeping the total mass of membrane protein constant; Fig. 4A) and also when using the corresponding isolated enzymes (Fig. 4B). The IC_50_ values in the membrane samples varied considerably in the nanomolar to micromolar (IACS-2858) or even millimolar (IACS-010759) ranges (Fig. 4A), with the mammalian enzymes inhibited most tightly, followed by *Y. lipolytica*, and with *P. denitrificans* complex I inhibited only very weakly. Similar trends were observed using the isolated complexes (Fig. 4B), with a general increase in IC_50_ values consistent with the higher hydrophobic phase volume present because of the detergent required for the isolated proteins, and the change of substrate [Q_6–10_ in membranes, decylubiquinone (dQ) for the isolated proteins]. Notably, however, the *Y. lipolytica* enzyme behaves more similarly to the mammalian complexes in the isolated state than in membranes. This discrepancy may reflect the different compositions of the membranes (phospholipid and protein) between species, factors that are not accessible to our structural analyses.

Sequence alignments of the regions of subunits ND1, NDUFS7, and NDUFS2 that form the ubiquinone-binding site are given in fig. S3. The main interaction partners of IACS-2858 identified in *M. musculus* (Glu24^ND1^, Arg25^ND1^, Gln32^ND1^, Arg34^ND1^, Phe224^ND1^, Tyr228^ND1^, Arg274^ND1^, Trp56^NDUFS7^, Asp80^NDUFS7^, and Arg87^NDUFS7^; Fig. 3A) are all conserved in *B. taurus* and *Y. lipolytica*, although Asp80^NDUFS7^(*Pd*-Glu, where *Pd* denotes the residue in *P. denitrificans*), Glu24^ND1^(*Pd*-Asp), Arg274^ND1^(*Pd*-Lys), Phe224^ND1^(*Pd*-Met), and Trp56^NDUFS7^(*Pd*-His) are not conserved in *P. denitrificans* (fig. S3). We thus extended the analysis to all residues within 5 Å of the IACS-2858 molecule in our structure (28 residues; see fig. S3).

1) Despite the differences in IC_50_ value observed for the two mammalian species, only two sequence differences were identified, at Met17^ND1^(*Bt*-Val) and Phe49^ND1^(*Bt*-Ile), where the residues in *B. taurus* are smaller, perhaps decreasing van der Waals interactions. In our IACS-2858–bound structure, Met17^ND1^ and Phe49^ND1^ are in close proximity to the 1,2,4-oxadiazole ring and trifluoromethoxy group, respectively, both of which are present in both inhibitors.

2) Consistent with its intermediate position in the affinity series, 10 residue differences were identified in *Y. lipolytica* (Fig. 4C and fig. S3), including Phe168^NDUFS2^(*Yl*-Leu), which makes hydrophobic interactions with the terminal sulfonylmethyl of IACS-2858, and Val85^NDUFS7^(*Yl*-Ile), adjacent to the inhibitor piperidine-phenyl N─C bond and with the possibility of steric clashes from the bulkier Ile.

3) In *P. denitrificans*, 20 residues within 5 Å of IACS-2858 differ relative to mouse complex I (fig. S3), including more substantial differences than identified for the eukaryotic enzymes. *Pd*-Glu(Asp80^NDUFS7^), one of the hydrogen-bonding triad in mouse complex I (Figs. 3A, a, and 4D, a), can no longer form a geometrically favorable hydrogen bond with Arg34^ND1^, disrupting the network of interactions at the sulfonylmethyl (Fig. 4D, a). *Pd*-Asp(Glu24^ND1^) can no longer reach the piperidine-*N* but is stabilized in its disconnected position by *Pd*-Lys(Arg274^ND1^) and *Pd*-Tyr(Leu28^ND1^) (Figs. 3A, b and 4D, b). The π-stacking partners of the 2-pyridone ring of IACS-2858, Trp56^NDUFS7^ and Phe224^ND1^, are replaced by His and Met and unable to maintain the π-stacking capacity (Fig. 3A, d and 4D, c). Furthermore, the nearby Leu55^ND1^ is mutated to Tyr, a similar change to the mutation to Phe in H292 cells that resulted in up to 70-fold loss of sensitivity to IACS-010759 (*10*) and perhaps inducing local rearrangement to minimize steric clashes (Fig. 4D, c). Last, Ala18^ND1^, in close proximity to the trifluoromethoxy terminus, is replaced by Ile, likely inducing steric clashes with the inhibitor molecule (Figs. 3B and 4D, d).

In summary, clear differences in residues surrounding the bound IACS-2858 in mouse and *P. denitrificans* provide straightforward explanations for the substantial difference in inhibition observed, and fewer differences for *Y. lipolytica* are consistent with its intermediate position in the series. The picture is less clear between the mammalian species. When clear inhibitor-protein contacts, such as hydrogen bonds or π-stacking interactions, are disrupted, it is easy to infer loss of affinity, but when the changes are between two hydrophobic residues of different sizes, the change in affinity is harder to predict: The change may increase steric clashes or decrease van der Waals interactions. A detailed understanding of how the inhibitor affinity is determined will require further structural and functional data in combination with site-directed mutagenesis to probe the effects of specific residue variation.

### Direction-dependent inhibition of electron transport

**Fig. 5:**
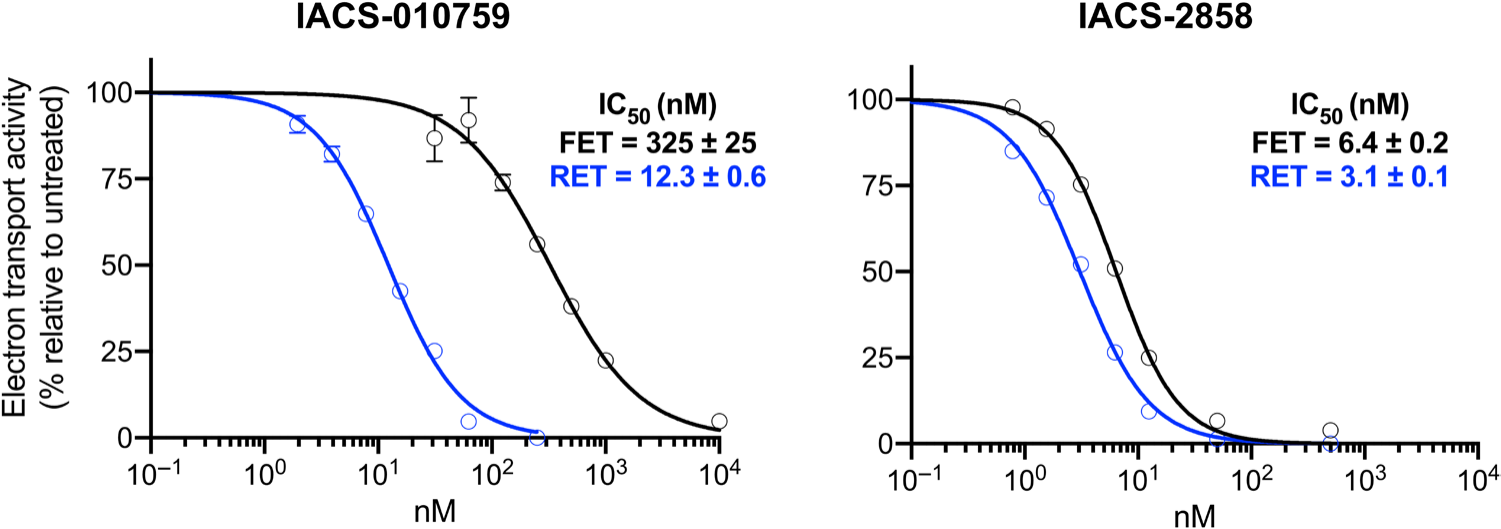
IACS-010759 and IACS-2858 inhibit RET more potently than FET. Experimentally measured FET (black) and RET (blue) rates (NADH oxidation rate and succinate oxidation–driven NAD^+^ reduction rates, respectively) of bovine SMPs (average ± SEM, *n* ≥ 3) are plotted against a titration of IACS-010759 (left) or IACS-2858 (right) concentrations. IC_50_ values are reported with the SE.

As IACS-010759 was previously reported to show different efficiencies for the inhibition of forward electron transport (FET) and RET (*34*), the inhibitory effects of IACS-010759 and IACS-2858 were investigated for both reactions using bovine heart submitochondrial particles (SMPs). Both compounds exhibited a direction-dependent inhibition of electron transfer, favoring the inhibition of RET over FET by more than 25-fold (IACS-010759) or 2-fold (IACS-2858) (Fig. 5) (note that IACS-2858 is a close-to-stoichiometric inhibitor even for FET, so that a 25-fold increase in potency would not be observed, even if present, in this case). Direction-dependent inhibition has been reported previously for the S1QEL family of complex I inhibitors (*5*). The chemical structures of the S1QELs resemble the structures of the compounds studied here (fig. S4), and they also inhibit RET more strongly than FET. Piericidin A has also been reported to inhibit RET more strongly than FET (*36*). In contrast, inhibition by the canonical inhibitor rotenone, which has a very different chemical structure, shows the opposite effect, inhibiting FET more strongly than RET (*37*). Although it is unclear whether the direction-dependent inhibition is relevant to the development of the IACS compounds as cancer drugs, the specific inhibition of RET, a focus for development of the S1QELs inhibitors, is relevant to, for example, the prevention of IR injury (*5*). Furthermore, the P25L-ND6 variant of mouse complex I has recently been demonstrated to be active only for FET, not RET (*38*). Although the mechanisms by which RET is prevented genetically or pharmacologically appear clearly distinct, combination of the two approaches now presents exciting opportunities to deconvolute and understand the roles of RET in pathophysiology.

### Kinetic evidence for the competitive mode of inhibitor binding

To investigate the kinetic inhibitor-binding mode of IACS-2858, proteoliposomes were created containing bovine complex I and varying concentrations of ubiquinone-10 across the *K_M_* curve (*17*, *23*). The complex I and ubiquinone-10 concentrations were defined relative to the hydrophobic phase volume (from the phospholipid concentration; see Materials and Methods), and the complex I concentration took into account the complex I orientation. The proteoliposomes were supplemented with the alternative oxidase (AOX) to recycle the ubiquinol that formed back to ubiquinone, and then their rates of steady-state NADH oxidation were determined in the presence of varying IACS-2858 concentrations. Basic reciprocal plot analyses (fig. S5) suggested the inhibition to be competitive and were not consistent with mixed, uncompetitive, or noncompetitive binding.

**Fig. 6:**
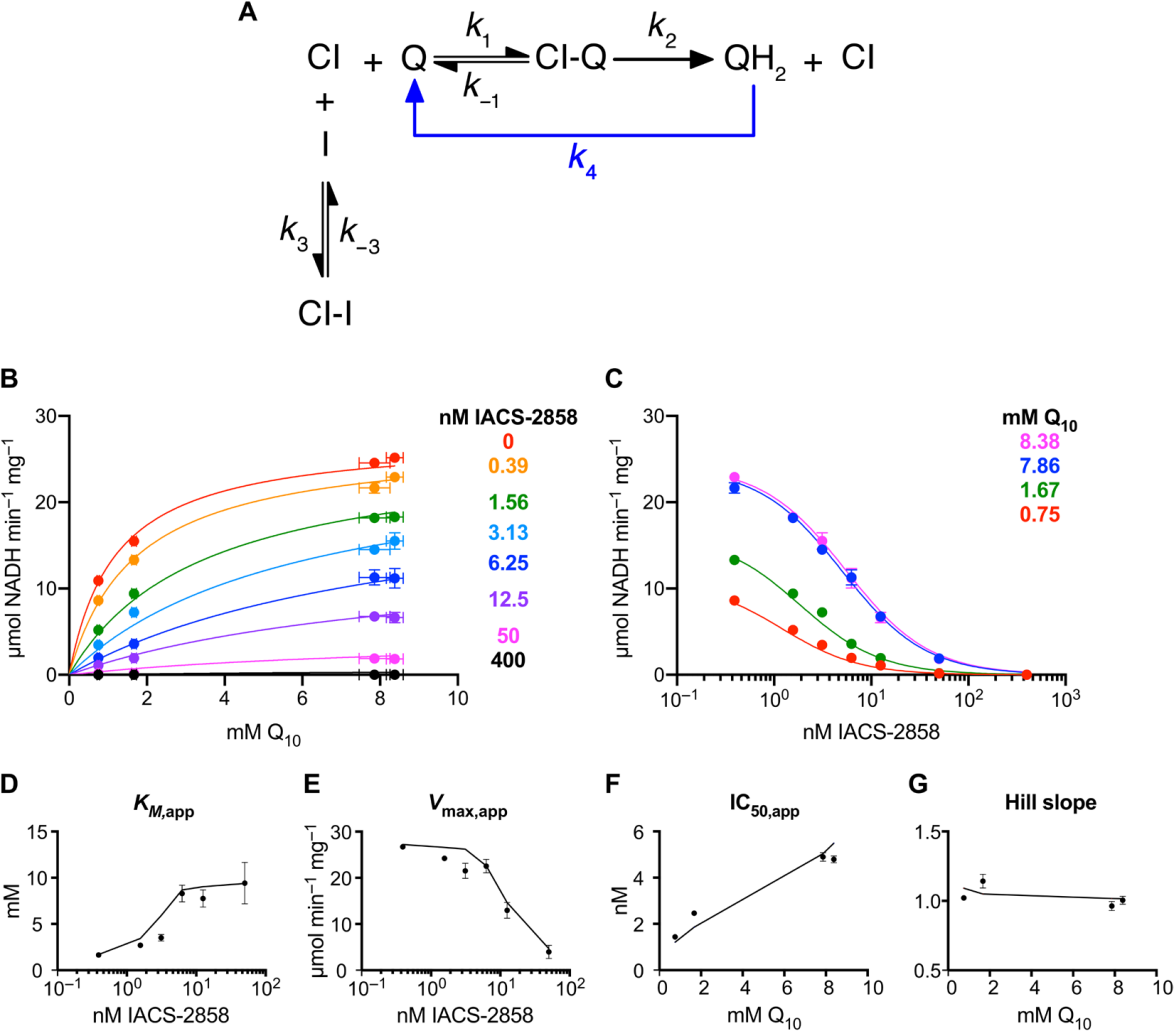
Competitive model for inhibition of complex I in proteoliposomes by IACS-2858. (**A**) Scheme for a simple competitive mode of inhibition. Rapid reoxidation of ubiquinol by AOX (*k*_4_) prevents appreciable levels accumulating. Experimentally measured rates (average ± SEM, *n* ≥ 3) are shown in (**B**) *K_M_* and (**C**) IC_50_ plots, alongside the best-fit predictions from the models (see table S3 for parameters). Plots of trends in (**D**) *K*_*M*,app_, (**E**) *V*_max,app_, (**F**) IC_50,app_, and (**G**) Hill slope were produced by using the Michaelis-Menten equation or the standard dose-effect relationship (see Materials and Methods) to fit the individual datasets shown in (B) and (C). Values from the experimental data are given as black points (error bars are 95% confidence intervals), and values from the output data from the models are shown as black lines.

To more conclusively characterize the mode of inhibition, comprehensive kinetic models were used to analyze the data, taking into account the tight binding nature of the inhibitor (the concentrations of the free and bound forms) and its partitioning between the membrane and aqueous phases (according to a calculated log_10_*P* of 5.12) (*23*). Consistent with the reciprocal plot evaluation, the data can be explained very well by the simplest competitive inhibition model (Fig. 6A): The sum of the squared residuals (SSR) for the differences between measured and calculated datapoints is low (Fig. 6, B and C, and table S3), and the trends in apparent *K_M_*, *V*_max_, IC_50_, and Hill slope values are reproduced well by the model (Fig. 6, D to G). Introducing an additional inhibitor-binding mode to create a mixed or a two-site competitive model improves the fit quality marginally (table S3) but not sufficiently to justify inclusion of an extra parameter, and these models are also not supported by our structural data. The classical uncompetitive binding model resulted in a poor fit (table S3). We conclude that the inhibitor binds competitively with ubiquinone-10: This simple model is supported by both our kinetic and structural data.

### Kinetic evidence for a single entrance to the channel

Given that the IACS-2858 binds only in the entrance to the channel, like a cork in a bottle, we used the inhibitor-bound enzyme as a basis to explore the possibility of additional entry pathways for short-chain, hydrophilic ubiquinone species. One such pathway, at the interface between subunits ND1, NDUFS7, NDUFS2, and NDUFA9, was tentatively proposed on the basis of site-specific affinity-labeling experiments (*39*). We thus tested for the reduction of ubiquinone-1 in the presence of IACS-2858, as it is small enough to fit into the active site, adjacent to Tyr108^NDUFS2^ at the top of the ubiquinone-binding channel, above the IACS-2858 molecule that plugs the lower section (Fig. 2C). No activity was observed in the presence of the inhibitor, arguing against a second entrance to the ubiquinone-binding active site, unless IACS-2858 binding occludes that entrance, too, by an allosteric mechanism.

### Global structure-activity relationships of noncanonical complex I inhibitors

Many noncanonical inhibitors of complex I, including BAY 87-2243 (*9*), mubritinib (*11*), carboxyamidotriazole (*11*), and S1QELs (*5*) (fig. S4), are composed, like IACS-010759 or IACS-2858, of “chains” of aromatic rings, suggesting a shared global structure-activity relationship. To investigate this hypothesis, we performed small-molecule conformational analyses on these compounds using the IACS-2858–bound cryo-EM model as a template. The compounds were flexibly aligned to the bound conformation of IACS-2858 and minimized in the binding site to generate a low-energy conformer for each. Local strain (the energy difference between the bound conformation and the nearest minimum) and global strain (the free energy associated with selecting the bioactive conformer from the solution-phase ensemble of unbound minima) were then calculated for each compound (table S4). The similar free energies across all these compounds with chains of aromatic rings (IACS-010759, BAY 87-2243, mubritinib, carboxyamidotriazole, S1QEL 1.1, and S1QEL 2.1) suggest that they are able to adopt a similar bioactive conformation or shape (table S4 and fig. S4) without incurring large free-energy penalties. Furthermore, each compound has one or more aromatic rings positioned where the π-stacking aromatic rings in IACS-2858 (2-pyridone and the adjacent phenyl ring) lie in the cryo-EM model, to engage in π-stacking interactions (fig. S4).

## DISCUSSION

Our structure of IACS-2858–bound complex I reveals a new mechanism of action for complex I inhibition, by an inhibitor that does not resemble the ubiquinone substrate, and highlights the significance of the π-stacking properties of the ubiquinone-binding tunnel. We show that IACS-2858 competes with ubiquinone for the ubiquinone-binding site, plugging its entrance like a cork in a bottle. The observed direction-dependent inhibitory efficiencies could be a consequence of the different abilities of ubiquinone and ubiquinol to compete against IACS-2858 for the binding site. It is likely that S1QELs occupy this same binding site as they structurally resemble IACS-2858 (fig. S4) and also show direction-dependent inhibitory efficiencies (*5*). Predominantly stabilized by π-stacking interactions with the surrounding aromatic and guanidino side chains and by hydrogen bonding, IACS-2858–mediated inhibition retains the active state of complex I. It does not cause any global or localized changes to the conformation of the substrate-binding cavity, as proposed previously by Tsuji *et al.* (*34*). The binding position of IACS-2858 also overlaps with a network of charged residues connected to a series of glutamates leading down into the membrane plane (the E-channel) (*19*–*21*), which may be important for energy conversion.

Our data show how cross-species sequence variations in the ubiquinone-binding site influence the binding of IACS-2858. The absence of dominating π-stacking partners in *P. denitrificans* almost completely abolishes IACS-2858 binding, while changes between hydrophobic residues of different sizes affect the inhibition observed for the mouse, cow, and yeast complexes. So far, differences between species of mammalian complex I have often been overlooked, as known IC_50_ values for canonical complex I inhibitors such as piericidin A or rotenone are conserved, whereas clear differences are often noted between mammalian, yeast, and bacterial species, such as for rotenone, which is reported to inhibit *Y. lipolytica* complex I more weakly than the mammalian enzyme (*40*). Here, our data illustrate how subtle differences in the ubiquinone-binding site within mammalian species can lead to at least a 10-fold variation in inhibition by an inhibitor that shows little resemblance to ubiquinone, suggesting that further pharmacologically relevant inhibitors may behave similarly. Our data and analyses are thus relevant to better understanding how complex I–targeted inhibitors interact with the human enzyme, to interpreting preclinical safety and efficacy analyses in animals, and also to identifying potential complex I–mediated off-target adverse consequences of drug therapy.

Increasing numbers of compounds identified as complex I–targeting drugs, such as BAY 87-2243 (*9*), mubritinib (*11*), carboxyamidotriazole (*11*), and S1QEL (*5*), are composed of linear “chains” of aromatic rings such as IACS-010759 and IACS-2858 and share a similar bioactive conformation (fig. S4). This general relationship acts in conjunction with the structure-activity relationships conferred by specific structural motifs, such as the heterocyclic 1,3-nitrogen motif found in mubritinib, carboxyamidotriazole (*11*), and IACS-010759, but not in the stronger-binding IACS-2858. Other enzymes with long “keyholes” also exhibit a similar mode of inhibition: human dihydroorotate dehydrogenase (DHODH; class 2), a mitochondrial enzyme that catalyzes ubiquinone-mediated oxidation of dihydroorotate to orotate in de novo pyrimidine biosynthesis, also exhibits a hydrophobic tunnel to the active site for FMNH_2_-ubiquinone redox catalysis (*41*). Several DHODH inhibitors such as the antiproliferative agents brequinar and atovaquone (*41*) and differentiation-inducing anticancer agent BAY 2402234 (*42*) are also composed of chains of aromatic rings and bind to the ubiquinone-binding site. All three inhibitors are involved in π-stacking interactions (off-center parallel or edge-to-face interactions) between at least one of their aromatic rings and a nearby aromatic residue, among other stabilizing interactions. Notably, the His-to-Ala mutation of the π-stacking partner of brequinar reduces the brequinar sensitivity by more than 100-fold in human DHODH (*41*, *43*). We thus propose that all the inhibitors of complex I formed by chains of aromatic rings are stabilized similarly by a permutation of π-stacking interactions with tunnel-lining residues, and bind in similar sites to IACS-2858, to block the entry of ubiquinone into the tunnel. Other hydrophobic complex I inhibitors with different chemical skeletons, such as the rotenoids, with three or more fused rings (*13*) still bind in the ubiquinone-binding site but in multiple binding locations, including in an overlapping site to IACS-2858 also stabilized by key π-stacking partners, Trp56^NDUFS7^ and Phe224^ND1^ (*26*).

Our observation of the importance of π-stacking interactions for IACS-2858 binding implies that the same principles may also help to govern ubiquinone binding. The density attributed to the aromatic rings of IACS-2858 overlays with the ubiquinone headgroups from ubiquinone-binding subsites adjacent to Trp56^NDUFS7^ and Phe220^ND1^ proposed from simulations (*44*–*46*), the second binding site of a ubiquinone-analog inhibitor piericidin A molecule next to Arg34^ND1^ proposed from simulations (*23*), and the aromatic headgroups of ligands modeled in recent cryo-EM structures, including the native substrate ubiquinone surrounded by Arg87^NDUFS7^, Phe220^ND1^, Phe224^ND1^, Tyr228^ND1^, Arg25^ND1^, Arg34^ND1^, and Arg274^ND1^ in *Y. lipolytica* (*35*), a ubiquinone-analog inhibitor DQA (*19*), and a DDM molecule (*25*) in *Y. lipolytica*. The close proximities of aromatic or guanidino side chains near the simulated or modeled headgroups and along the ubiquinone-binding site (Phe56^ND1^, Phe49^ND1^, Phe220^ND1^, Phe224^ND1^, Tyr228^ND1^, Trp56^NDUFS7^, Arg87^NDUFS7^, Arg25^ND1^, Arg274^ND1^, Arg279^ND1^, Arg34^ND1^, Phe168^NDUFS2^, Phe167^NDUFS2^, Phe86^NDUFS7^, and His59^NDUFS2^; fig. S6A) and the proposed “staging posts” mechanism of ubiquinone transit (*44*–*46*) imply a stepwise π-stacking pathway that guides the ubiquinone headgroup along the tunnel to the site of redox catalysis, and that the ubiquinone-binding subsites identified from simulations and cryo-EM structures represent intermediary positions corresponding to minima in the free-energy profile. This model may further help to explain why the *V*_max_ and *k*_cat_ values of ubiquinone-1 and ubiquinone-2 are substantially lower than for ubiquinone-10 (*17*), as their shorter chain lengths make them vulnerable to getting “trapped” at intermediate “π-stacking steps.” Moreover, the predicted positions of isoprenoids 2 to 7, 9, and 10 of the ubiquinone-10 tail (*17*) are within π-stacking distances with aromatic residues or guanidino groups of arginines lining the channel (fig. S6B).

On the basis of site-specific chemical modification experiments using photoreactive inhibitors and ligand-directed tosylate chemistry (*39*) to probe the ubiquinone-binding site, Miyoshi and co-workers (*39*) have proposed an “open” binding area for ubiquinone/inhibitors that does not encompass the current access channel predicted from structural studies (*18*–*20*, *23*, *25*, *35*) and with the inhibitor-binding sites distributed around the structure-predicted channel (*39*). The authors have also proposed a different entrance to their “open” binding pocket (in the interface between the hydrophilic and hydrophobic domains) and that the channel undergoes large structural rearrangements to form an “open pathway” and allow a wide range of ligands access to the interior (*39*). However, our cryo-EM structure of complex I in the IACS-2858–bound state and our kinetic data on the lack of NADH:ubiquinone-1 oxidoreduction activity in the presence of IACS-2858 argue against this proposed alternative entrance, unless IACS-2858 allosterically constrains or blocks the proposed second entrance. Furthermore, photoaffinity-labeling experiments performed on IACS-010759 suggested that the inhibitor does not occupy the proposed ubiquinone-binding tunnel but rather binds to the region under the ND1 TMH5-TMH6 loop (*34*), in contradiction to our IACS-2858–bound structure.

In summary, our IACS-2858–bound structure and kinetic studies define how inhibitors with chains of aromatic rings inhibit catalysis by complex I by a cork-in-bottle mechanism. Our data provide a basis to guide future biorational drug design of complex I inhibitors and further our understanding of substrate binding and inhibitor binding mediated by the channel-lining π-stacking residues that comprise the keyhole in the keyhole-lock-key model for enzyme catalysis.

## MATERIALS AND METHODS

### Chemical synthesis of IACS-2858 and IACS-010759

IACS-010759 was synthesized as described in Molina *et al.* (*10*). The synthesis of IACS-28258 is described below.

#### Step 1


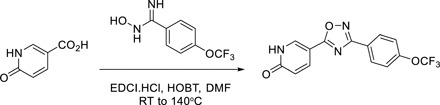


##### 5-(3-(4-(Trifluoromethoxy)phenyl)-1,2,4-oxadiazol-5-yl)pyridin-2(1H)-one

A mixture of 6-oxo-1,6-dihydropyridine-3-carboxylic acid (180 mg, 1.3 mmol), *N*-ethyl-*N*′-(3-dimethylaminopropyl)carbodiimide (EDCI).HCl (200 mg, 1.3 mmol), hydroxybenzotriazole (HOBT) (200 mg, 1.6 mmol), and *N*-hydroxy-4-(trifluoromethoxy)benzimidamide (300 mg, 1.3 mmol) in *N*,*N*′-dimethylformamide (DMF; 13 ml) was stirred at room temperature (RT) for 8 hours and then at 140°C for an additional 2 hours. The reaction mixture was diluted with EtOAc, washed with H_2_O, and concentrated under reduced pressure. The residue was purified by SiO_2_ gel chromatography (20% EtOAc in hexane, 20 to 100% EtOAc) to afford the title compound as a white solid (110 mg, 26%). Mass spectrometry (electrospray ionization) [MS (ES^+^)] C_14_H_8_F_3_N_3_O_3_ requires: 323, found: 324 [M + H]^+^.

#### Step 2


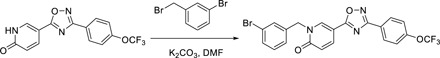


##### 1-(3-Bromobenzyl)-5-(3-(4-(trifluoromethoxy)phenyl)-1,2,4-oxadiazol-5-yl)pyridin-2(1*H*)-one

To a suspension of 5-(3-(4-(trifluoromethoxy)phenyl)-1,2,4-oxadiazol-5-yl)pyridin-2(1*H*)-one (200 mg, 0.619 mmol) and K_2_CO_3_ (128 mg, 0.928 mmol) in DMF (16 ml) was added 1-bromo-3-(bromomethyl)benzene (186 mg, 0.743 mmol). The mixture was stirred at RT for 1 hour, diluted with water (5 ml), and extracted with EtOAc (3 × 5 ml). The combined organic layers were washed with water (4 × 5 ml) and brine (5 ml), dried over Na_2_SO_4_, and concentrated under reduced pressure. The residue was purified by SiO_2_ gel chromatography (0 to 40% EtOAc/hexane) to provide the title compound as an off-white solid (220 mg, 72%): MS (ES^+^) C_21_H_13_BrF_3_N_3_O_3_ requires: 492, found: 493 [M + H]^+^.

#### Step 3


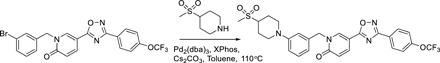


##### 1-(3-(4-(Methylsulfonyl)piperidin-1-yl)benzyl)-5-(3-(4-(trifluoromethoxy)phenyl)-1,2,4-oxadiazol-5-yl)pyridin-2(1*H*)-one

To a suspension of Cs_2_CO_3_ (36 mg, 0.11 mmol), XPhos (4.8 mg, 5.1 μmol), tris(dibenzylideneacetone)dipalladium(0) (4.7 mg, 6.09 μmol), and 1-(3-bromobenzyl)-5-(3-(4-(trifluoromethoxy)phenyl)-1,2,4-oxadiazol-5-yl)pyridin-2(1*H*)-one (25 mg, 0.051 mmol) in toluene (0.50 ml), previously degassed with N_2_, was added 4-(methylsulfonyl)piperidine (8.0 mg, 0.051 mmol). The mixture was sealed in a vial, heated to 110°C for 2 hours, then cooled to RT, filtered through a pad of Celite, and concentrated under reduced pressure. The residue was purified by prep–high-performance liquid chromatography (HPLC) [mobile phase: A = 0.1% trifluoroacetic acid (TFA)/H_2_O and B = 0.1% TFA/MeCN; gradient: B = 30 to 70% in 12 min; column: C18] to give the title compound MS (ES^+^) C_27_H_25_F_3_N_4_O_5_S requires: 574, found: 575 [M + H]^+^; ^1^H nuclear magnetic resonance (600 MHz, CDCl_3_) δ 8.35 (s, 1H), 8.14 (d, *J* = 8.4 Hz, 2H), 8.03 (d, *J* = 9.6 Hz, 1H), 7.34 (d, *J* = 8.4 Hz, 2H), 7.31 (m, 1H), 7.04 (s, 1H), 7.97 (dd, *J* = 8.4 Hz, 2.4 Hz, 1H), 6.91 (d, *J* = 8.4 Hz, 1H), 6.77 (d, *J* = 9.6 Hz, 1H), 5.20 (s, 2H), 3.86 (m, 2H), 2.99 (m, 1H), 2.87 (s, 3H), 2.85 (m, 2H), 2.29 (m, 2H), and 2.02 (m, 2H).

### Preparation of mouse complex I inhibited by IACS-2858 for cryo-EM

Complex I inhibited by IACS-2858 was prepared by a method adapted from that of Bridges *et al.* (*23*) for preparing mouse complex I inhibited by piericidin A1. C57BL/6 mice were euthanized by cervical dislocation in accord with the UK Animals (Scientific Procedures) Act 1986 (PPL: P6C97520A, approved by the local ethics committee and the UK Home Office), and mitochondrial membranes were prepared from heart tissue as described previously (*20*). Starting from membranes containing ~40 mg of total protein, the proteins were solubilized from the membranes by addition of 1% DDM (Glycon), and the mixture was centrifuged. Then, the detergent-solubilized complexes were separated by ion-exchange chromatography using three 1-ml Hi-Trap Q Hp columns (GE Healthcare) connected in series. The complex I–containing fractions were pooled and concentrated to 100 μl using a 100-kDa molecular weight cutoff (MWCO) Amicon Ultra concentrator (Merck Milipore Ltd.). Ten microliters was removed as a control sample, and the remaining 90 μl was added to a glass vial containing 2.15 mg of IACS-2858 (dried down from a chloroform stock solution). The level of inhibition achieved was determined by comparing the initial rates of catalysis by the control and inhibited samples in buffer containing 20 mM tris-HCl (pH 7.5 at 32°C), 0.15% soy bean asolectin (Avanti Polar Lipids), and 0.15% CHAPS (Merck Chemicals Ltd.), using 200 μM dQ, with catalysis initiated by 200 μM NADH and monitored at 340 to 380 nm (ε = 4.81 mM^−1^ cm^−1^). The level was deemed acceptable if catalysis was inhibited by >80%. Then, the test and control samples were applied separately to a Superose 6 increase 5/150 column (GE Healthcare) and eluted in 20 mM tris-HCl (pH 7.14 at 20°C), 150 mM NaCl, and 0.05% DDM. The concentration of the peak fraction for the inhibited protein, eluting at ~1.65 ml, was estimated using a NanoDrop ultraviolet-visible spectrophotometer [ε_280_ = 0.2 (mg ml^−1^)^−1^], and the level of inhibition of the final eluted IACS-2858–treated sample was determined as above. For the sample subjected to cryo-EM analysis, the concentration was 7.1 mg ml^−1^, the level of inhibition was 81.3 ± 4.7%, and the specific activity of the control protein was 10.4 ± 0.2 μmol min^−1^ mg^−1^.

### Cryo-EM grid preparation and image acquisition

UltrAuFoil gold grids (0.6/1, Quantifoil Micro Tools GmbH) were prepared as described previously (*20*, *22*). Briefly, they were glow-discharged (20 mA, 90 s), incubated in a solution of 5 mM 11-mercaptoundecyl hexaethyleneglycol (TH 001—m11.n6-0.01, ProChimia Surfaces) in ethanol for 2 days in an anaerobic glovebox, then washed with ethanol, and dried just before use. Using a Vitrobot Mark IV (FEI), 2.5 μl of IACS-2858–bound complex I solution (7.1 or 5.5 mg ml^−1^; from the same preparation) were applied to the grids before blotting for 10 s at a force setting of −10, at 100% relative humidity and 4°C, and then plunge-frozen into liquid ethane. Six grids were screened for particle number and distribution, and two grids, one frozen at each concentration, were selected. They were imaged using a Gatan K2 detector and GIF Quantum energy filter mounted on an FEI 300-keV Titan Krios microscope (Thermo Fisher Scientific) with a 100-μm objective aperture and EPU software at The Nanoscience Centre, University of Cambridge. The energy filter was operated in zero-energy-loss mode with a slit width of 20 eV. Data were collected at 1.074 Å pixel^−1^ (×130,000 nominal magnification) with a defocus range of −1.5 to −2.7 μm, and the autofocus routine ran every 10 μm. The dose rates for three separate data collections, datasets 1, 2, and 3, were 5.87, 5.84, and 5.78 electrons Å^−2^ s^−1^, respectively, with 10-s exposures captured in 25 frames. The total dose was thus ~50 electrons Å^−2^ in each case.

### Cryo-EM data processing

All data were processed using two different versions of RELION, 3.0.7 and 3.1 (*47*), unless stated otherwise. In RELION 3.0.7, MotionCor2 1.2.1 (*48*) (with dose weighting) was used to correct for beam-induced movement, and CTFFIND-4.1 was used for contrast transfer function (CTF) estimation. Following autopicking (using a general network model without training) and manual curation on crYOLO-1.3.5 (*49*), 103,974 particles were extracted from 1927 micrographs in dataset 1, 42,849 from 530 micrographs in dataset 2, and 114,998 from 1454 micrographs in dataset 3. The particles were subjected to two-dimensional (2D) classification on cryoSPARC v2.5.0 (*50*) (dataset 1) and RELION 3.0.7 (datasets 2 and 3) where 83,709, 41,683, and 110,764 particles were retained, respectively, and then to two rounds of 3D classification in RELION 3.0.7 over five classes with angular sampling down to 0.2° (fig. S1). The particles in the major classes in datasets 1, 2, and 3 (19,017, 4856, and 28,382 particles, respectively) were reextracted and subjected to iterative rounds of CTF refinement, including beam tilt and per-particle astigmatism correction, and Bayesian polishing. The three datasets were then combined (52,255 particles) and subjected to 3D classification into two classes with angular sampling down to 0.1°; 44,000 particles were retained in the major class (fig. S1). To take advantage of the improvements available in RELION 3.1, raw micrographs from the three datasets were categorized into three optics groups, and RELION’s implementation of motion correction (with dose weighting) was applied. The datasets were then merged and CTFFIND-4.1 CTF correction applied. The final 44,000 particles from RELION 3.0.7 were imported into RELION 3.1, extracted, and then subjected to iterative rounds of CTF refinement, including anisotropic magnification estimation, per-particle defocus, astigmatism and B-factor fittings, and beam tilt corrections (three- and fourfold astigmatism) per optics group. Each optics group was individually subjected to Bayesian polishing, and the subsequent shiny particles were recombined and CTF-refined, before the final 3D refinement was performed with solvent-flattened Fourier shell correlation (FSC). The map was postprocessed using default parameters, and a mask was created using the molmap command on a near-complete model in UCSF ChimeraX (*51*) alongside mask creation tools in RELION, with a soft edge of seven pixels and no extension. The mask thus excludes the detergent belt, so the resolution statistics refer only to the protein densities. The global resolution of the final map was 3.0 Å, based on the FSC = 0.143 criterion. Last, pixel sizes were adjusted to three decimal places (1.055 Å), determined by refining the spherical aberration and by comparison to existing density maps of mouse complex I in the active and piericidin A1–bound states (*23*) using the “fit in map” function in UCSF ChimeraX (*51*). Local resolution (fig. S2A) was calculated using RELION 3.1 and the MonoRes implementation in Scipion 1.2.1 (*52*, *53*) and visualized in UCSF ChimeraX (*51*).

### Model building, refinement, and validation

A working model for the active mouse complex I (PDB: 6ZR2) (*23*) was rigid body–fitted into the map using UCSF ChimeraX (*51*). The model was then refined against the RELION-sharpened map by cycles of manual adjustment in Coot 0.9-pre (*54*) and real-space refinement in Phenix 1.16-3549 (*55*) with secondary structure restraints. The IACS-2858 molecule was created using Coot’s ligand builder Lidia, and its geometry restraints were generated using Phenix eLBOW. The inhibitor molecule was manually fitted into its electron density identified in the map and then merged with the model in Coot and real space refined in Phenix. The model statistics (table S1) were produced by Phenix, MolProbity 4.4, and EMRinger (score of 4.00). Model-to-map FSC curves were generated using the Phenix Comprehensive validation (cryo-EM) tool. Last, the model was checked for overfitting (fig. S2B) by randomizing the atom coordinates by a mean value of 0.5 Å using Phenix PDB tools (*55*) and then refining it against one of the two unsharpened unfiltered half maps. A model-to-map FSC curve was then calculated between the refined model and the half map that it was refined against (FSC_work_), and a cross-validated FSC was calculated between the refined model and the other half map not used for refinement (FSC_free_) (*56*).

### Comparisons of cryo-EM maps and models

Map-to-map real-space correlations between the IACS-2858–bound map and the active (*23*) or deactive (EMD: 4356) (*20*) maps were performed using the “fit in map” function in Chimera. Contour levels were manually adjusted to 0.055 (IACS-2858), 0.031 (active), and 0.082 (deactive). All-atom RMSD calculations between the IACS-2858–bound model and the active (PDB: 6ZR2) or deactive (PDB: 6G72) models were performed using the Align command in PyMOL.

### Identification of enzyme-inhibitor contacts

Atomic contacts (direct interactions including polar and nonpolar as well as favorable and nonfavorable interactions) between IACS-2858 and its neighboring residues in the model were identified using the contacts and clashes commands in UCSF ChimeraX (*51*) and the default cut-off value for the van der Waal’s overlap (defined as the sum of their van der Waal’s radii minus the distance between their centers) of ≥−0.4 Å. Hydrogen-bonding partners were identified using the H-bond tool in UCSF ChimeraX (*51*), for which the geometric criteria are based on a survey of small-molecule crystal structures.

### Quinone cavity determination

The interior surface of the ubiquinone-binding channel was predicted using CASTp (*57*), which computes a protein surface topology from a PDB model. The default 1.4-Å radius probe was used, and the results were visualized in PyMOL using the CASTpyMOL 3.1 plugin and by UCSF ChimeraX (*51*).

### Preparation and characterization of proteoliposomes

Complex I from bovine heart were prepared and used to make proteoliposomes as described previously (*17*, *23*, *58*). Briefly, liposomes were formed from 8 mg of phosphatidylcholine, 1 mg of phosphatidylethanolamine, and 1 mg of cardiolipin (bovine heart extracts from Avanti Polar Lipids), together with varying amounts of ubiquinone-10 (Sigma-Aldrich), in ~800 μl of 10 mM 3-morpholinopropane-1-sulfonic acid (Mops)-KOH (pH 7.5) and 50 mM KCl. Following extrusion through a 100 nm of Nuclepore polycarbonate membrane (Whatman), the liposomes were partially solubilized with 0.5% sodium cholate (Anatrace), and 0.2 mg of complex I was added for reconstitution. Detergent was then removed using a PD-10 desalting column (GE Healthcare). The proteoliposomes were collected by centrifugation, resuspended, and flash-frozen in liquid nitrogen for storage at −80°C. Complex I concentration and orientation were quantified using the NADH:APAD^+^ activity assay as described previously (*17*, *23*, *58*). Phospholipid phase volumes were calculated as described previously (*17*, *23*, *58*) to determine the amount of phospholipid present and by assuming that 1 mg of phospholipid occupies ∼1 μl. Ubiquinone-10 contents were quantified by HPLC, by reference to a set of standard samples, using a Nucleosil 100-5C18 column and a Dionex Ultimate 3000 RS electrochemical detector as described previously (*17*, *23*, *58*). Ubiquinone-10 concentrations were defined relative to the phospholipid phase volume.

### IACS-2858 inhibition kinetics

All catalytic activity assays were conducted at 32°C in 96-well plates using a Molecular Devices Spectramax 384 plus plate reader. Catalysis was initiated by addition of 200 μM NADH and monitored at 340 and 380 nm (ε_340–380_ = 4.81 mM^−1^ cm^−1^). Linear rates were measured for all assays, inhibitors were added from dimethyl sulfoxide stock solutions as required, and inhibitor-insensitive rates (using either 1 μM piericidin A or 1.4 μM IACS-2858) were subtracted from each measured rate. Membrane samples were prepared as described previously (*17*, *20*, *59*, *60*), diluted to 20 μg ml^−1^ in 10 mM tris-SO_4_ (pH 7.5) and 250 mM sucrose, and supplemented with 3 μM horse heart cytochrome c (except for *P. denitrificans*, for which the activity is unaffected by the addition). Mouse, bovine, and yeast (*Y. lipolytica*) complex I were purified as described previously (*17*, *20*, *59*); diluted to 0.5 nM in 20 mM tris-HCl (pH 7.5), 0.15% asolectin, and 0.15% CHAPS; and assayed using either 200 μM dQ or 200 μM ubiquinone-1 (Sigma-Aldrich). Complex I was purified from *P. denitrificans* strain Pd1222 (*61*) and provided by O. D. Jarman (MRC Mitochondrial Biology Unit, University of Cambridge); it was assayed likewise but in 10 mM MES-HCl (pH 6.5), 25 mM NaCl, and 2 mM CaCl_2_. Bovine SMPs were prepared as described previously (*61*) and diluted to 20 μg ml^−1^ in 10 mM tris-SO_4_ (pH 7.5) and 250 mM sucrose for FET and RET assays. The RET assay was initiated using 1 mM NAD^+^, 1 mM ATP, 10 mM succinate, 1 mM cyanide, and 2 mM MgSO_4_, in the absence of NADH. AOX from *Trypanosoma brucei brucei* was prepared as described previously (*23*). Complex I proteoliposomes were assayed using 10 mM Mops-KOH (pH 7.5), 50 mM KCl, and AOX (10 μg ml^−1^). Kinetic data were fit to the standard dose-effect relationship [activity (%) = 100 / (1 + (IC_50_) / ([inhibitor])^Hill slope^] using GraphPad Prism version 8.0.0, and IC_50_ values are reported with their SEs. Note that IC_50_ values are specific to the assay system described. The values presented are therefore comparable for a given system (for example, isolated complex I) but not between systems (for example, isolated complex I and membranes).

Proteoliposome data were modeled as described previously (*23*) using the ordinary differential equation solver, ode15s, in MATLAB (MathWorks, R2017a) with all the reverse rate constants (*k*_−1_, *k*_−3_, etc.) set to 1. Rate calculations were terminated once they reached steady state (judged as a change in an enzyme-substrate complex concentration of <1 pM s^−1^ upon each simulated timestep). The overall rate of reaction (NADH oxidation) was calculated as the product of the steady-state concentration of enzyme-substrate complex and the turnover rate (*k*_2_) and then expressed as a specific activity. Fitting was repeatedly initiated with a random set of values for the parameters to be varied and targeted on the parameter combination with the smallest error between the calculated and measured rates; the data were modeled simultaneously over all ubiquinone-10 and IACS-2858 concentrations. The log_10_*P* value of IACS-2858 (5.12) was calculated using MarvinSketch Version 16.5.2.0, Consensus Model, ChemAxon, MA (*62*). For the final models, bootstrapping of the residuals (*n* = 1000) was conducted to derive fitting statistics (mean, median, 95% confidence intervals, and SSRs) of model parameters.

### Small-molecule conformational analyses

The structure model of mouse complex I with IACS-2858 bound was prepared, protonated using the Structure Preparation workflow, and then minimized with tethered restraints, using MOE (Molecular Operating Environment; 2019). A selection of noncanonical complex I inhibitors were flexibly aligned to the fixed, bound conformation of IACS-2858 in multiple orientations and minimized in the binding site, and then the best conformer was selected by comparing the protein-ligand interaction energies calculated using the AMBER10:EHT force field in MOE. Local and global strain energies for these conformers were calculated using FreeForm, part of the SZYBKI software package (SZYBKI 2.2.0.4) (*63*), using the -track option.

## SUPPLEMENTARY MATERIALS

Supplementary material for this article is available at http://advances.sciencemag.org/cgi/content/full/7/20/eabg4000/DC1

## Appendix

View/request a protocol for this paper from *Bio-protocol*.
